# Port site parasitic leiomyoma after laparoscopic myomectomy: a case report and review of the literature

**DOI:** 10.1186/s13256-018-1873-y

**Published:** 2018-11-15

**Authors:** Felix Mwembi Oindi, Steve Kyende Mutiso, Timona Obura

**Affiliations:** Department of Obstetrics and Gynaecology, Aga Khan University, P.O. Box 30270-00100, Nairobi, Kenya

**Keywords:** Laparoscopic myomectomy, Morcellation, Parasitic leiomyoma

## Abstract

**Background:**

Uterine fibroids are the commonest benign gynecological tumors. Laparoscopic myomectomy is becoming increasingly popular as one of the surgical treatment options for symptomatic cases. Large tissues such as leiomyomas or even the uterus need to be morcellated in order to be retrieved from the abdominal cavity. Some of the morcellated fragments or small fibroids may be accidentally left in the abdominal cavity during the retrieval process. These may subsequently become implanted in the abdominal cavity, develop blood supply from the surrounding structures, and grow to form parasitic myomas with varied clinical presentation, depending on the location and size.

**Case presentation:**

A 47-year-old African woman presented to our hospital 6 years after laparoscopic myomectomy with a lower abdominal mass. Her work-up revealed an anterior abdominal wall mass consistent with uterine leiomyoma. She was scheduled for excision of the mass, which was subsequently histologically confirmed to be a uterine fibroid.

**Conclusions:**

Parasitic leiomyomas are a rare late complication of power morcellation following laparoscopic myomectomy or hysterectomy. Most patients present with an abdominal/pelvic mass and may need surgical excision to relieve the symptoms. Care should be taken during power morcellation to prevent excessive fragmentation of the tissues, some of which may become implanted and persist to form parasitic myomas. Moreover, effort should be made to retrieve all myoma fragments by carefully checking the abdominal cavity. Whenever possible, the morcellation should be done in a containment bag.

## Background

Uterine fibroids are the most common benign gynecological tumors of the female genital tract. Despite their relatively high prevalence, clinically apparent in up to 40% of women over 40 years of age, the majority of the patients with uterine fibroids are asymptomatic [1, 2]. Various treatment modalities have been employed for symptomatic patients, ranging from medical to surgical interventions. Surgical management may be performed through laparotomy or minimally invasive surgery, among others [1, 3].

The increasing availability of power morcellators has made it possible for large leiomyomas to be removed laparoscopically with the benefit of decreased blood loss, shorter hospital stay, and faster recovery time [2, 4, 5]. Other options for tissue retrieval, such as through a colpotomy incision or through a minilaparotomy, are becoming less popular [2]. The fragmentation of the fibroids using the morcellator may lead to peritoneal seeding, which, if not detected at the time of the operation, may grow to form parasitic leiomyomas [1, 2]. This is a rare late complication of laparoscopic myomectomy [6]. We present a case of a 47-year-old woman who presented with a left port site leiomyoma recurrence after laparoscopic myomectomy with power morcellation, and we review the current literature on myoma recurrence after laparoscopic myomectomy.

## Case presentation

A 47-year-old, para 3 + 0, of African descent presented with a lower abdominal mass that had been progressively increasing over a 3-year period. The mass was on her left lower abdomen and was not painful. Six years earlier, she had undergone laparoscopic myomectomy with power morcellation for myoma retrieval.

She also reported persistent menorrhagia and dysmenorrhea since the previous surgery, for which she was mainly receiving medical therapies. These included tranexamic acid and mefenamic acid with the onset of menses, which provided good symptom relief. She reported no symptoms of anemia, such as easy fatigability, palpitations, or dyspnea, and had not used any hematinics prior to the current presentation. Her Papanicolaou smears for cervical cancer screening were up-to-date with normal cytology.

All her three deliveries were through cesarean section, two before and one after the initial surgery. She had no urinary symptoms such as frequency of micturition, dysuria, or incomplete voiding. In addition, she had no bowel symptoms such as constipation.

**Fig. 1:**
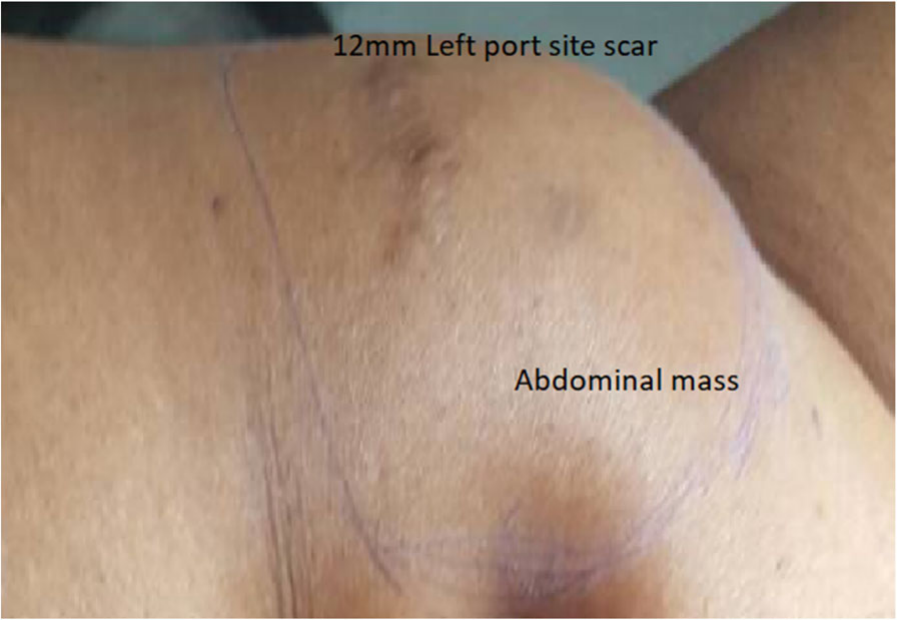
Lower anterior abdominal wall mass at the previous left laparoscopic myomectomy port site

The examination revealed an anterior abdominal wall mass in the left iliac fossa. The mass was firm, mobile, and nontender overlying the previous left laparoscopy port site (Fig. 1). This was the site used for morcellation during the previous laparoscopic myomectomy. In addition, she had a bulky 16-week fibroid uterus.

**Fig. 2:**
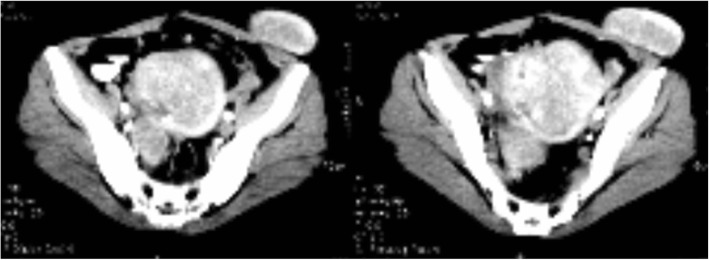
Hyperintense T2-weighted magnetic resonance imaging studies showing the parasitic left iliac fossa anterior abdominal wall myoma with cystic degeneration

Her preoperative work-up included a pelvic ultrasound examination, which revealed multiple intramural fibroids with a superficial left hypoechoic mass in the left iliac fossa region measuring 4 cm by 3 cm. Abdominopelvic magnetic resonance imaging (MRI) revealed an anterior abdominal mass measuring 7.2 cm by 5.1 cm by 3.4 cm on the anterior abdominal wall in the left iliac fossa (Fig. 2). A full hemogram revealed a hemoglobin level of 11.8 g/dl, white blood cell count of 4.2 × 10^9^/L, and platelet count of 261,000 × 10^9^/L.

**Fig. 3:**
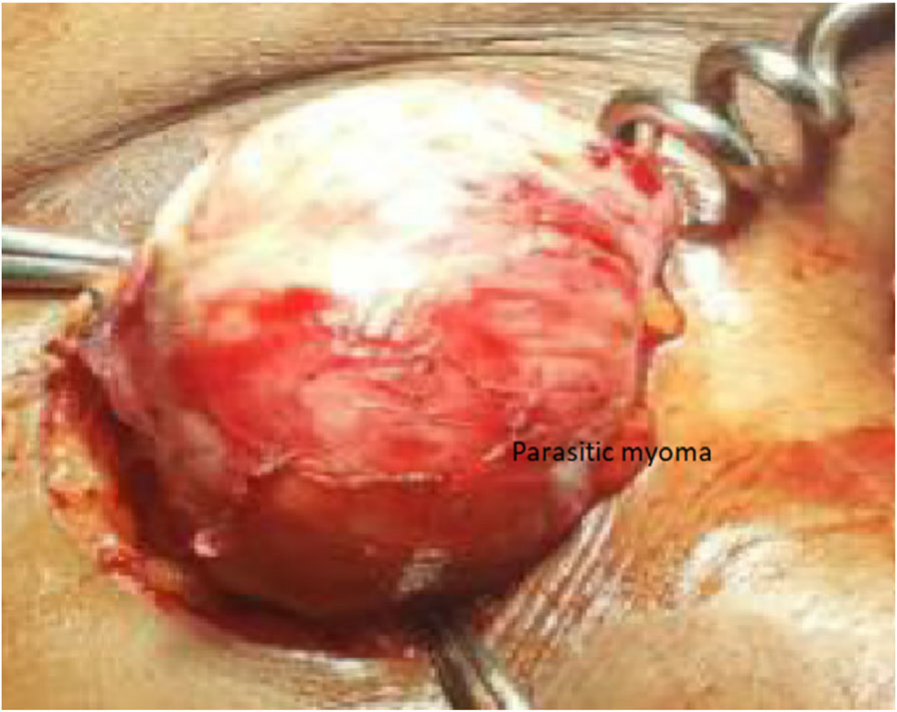
Parasitic myoma at the previous 12-mm left laparoscopy port site before excision

**Fig. 4:**
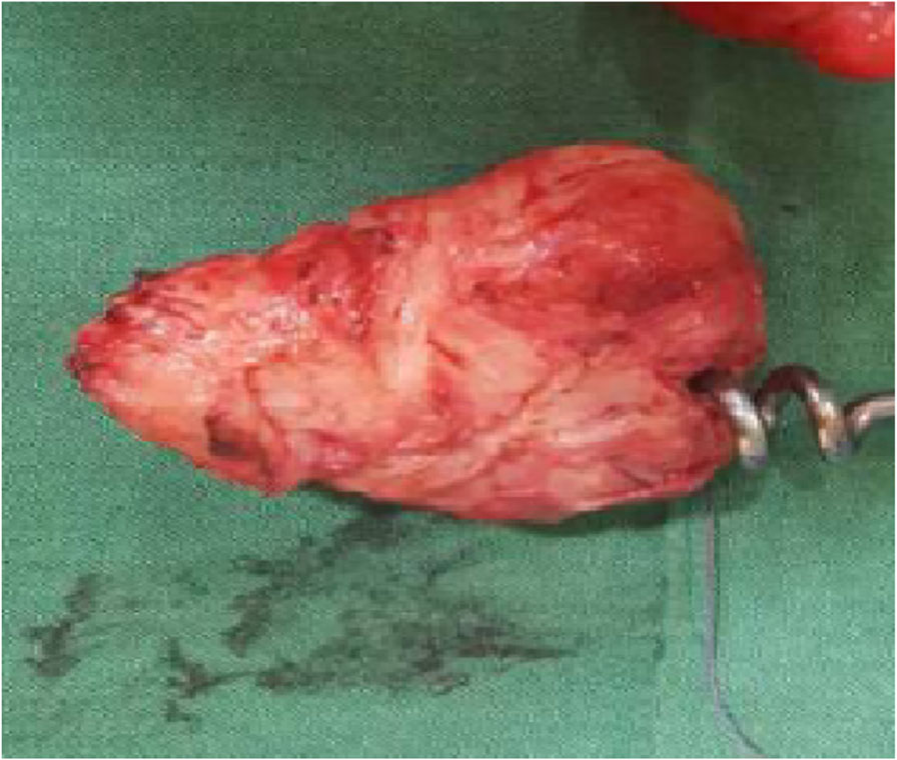
Parasitic myoma after excision

Management options were discussed, and the patient opted to have a total abdominal hysterectomy and excision of the abdominal mass. The hysterectomy was done through the previous cesarean section scar site. A separate 5-cm incision was made over the left iliac fossa via the previous 12-mm port site scar, and dissection was done to retrieve a 7.5-cm by 5.5-cm by 3.5-cm parasitic myoma (Figs. 3 and 4). The parasitic myoma was inaccessible through the previous cesarean section scar, necessitating a separate incision. In addition, the parasitic myoma was attached to the subcutaneous tissue with no visceral or major vascular attachments. The histology of the mass showed interlacing bundles of benign smooth muscle fibers consistent with a leiomyoma. The patient’s postoperative recovery was uneventful, and her review 6 months following the surgery was unremarkable.

## Discussion

Laparoscopic myomectomy and hysterectomy are increasingly becoming more available. To aid in tissue removal, large tissues such as the uterus and leiomyomas are often fragmented with the use of power morcellators. Some of the resultant small fragments may inadvertently remain in the abdominal cavity and become implanted with development of blood supply, resulting in subsequent growth [7–9]. These parasitic leiomyomas form a rare late sequela of laparoscopic myomectomy [10, 11] with an incidence of 0.2–1.25% and a median diagnosis interval of 48 months [1, 2, 5]. Our patient first noted the abdominal mass at about 36 months after her laparoscopic myomectomy with eventual confirmation at about 72 months. Despite better patient outcomes following minimally invasive surgery, caution should be applied to prevent the spread of occult sarcomas, which would significantly lower patient survival [12–14].

The clinical presentation is quite nonspecific and depends on the site of recurrence. The common sites include the pelvic cavity/wall, small intestines, rectum, cecum, vaginal/cervical stump, and laparoscopic port site [5, 7, 10]. Most patients will be asymptomatic with an incidental finding of the parasitic myoma during other investigations or procedures, especially if small [2, 8]. However, when symptomatic, the most common symptoms include abdominal/pelvic pain, abdominal mass or pressure, abdominal distention, and vaginal bleeding [2, 10]. Our patient presented with a progressively increasing abdominal mass. The absence of pain and pressure symptoms on the bladder or bowel may have contributed to a delay in her presentation.

Given the nonspecific presentation, there is no specific investigation that is ideal in the diagnosis of parasitic myomas. The definitive diagnosis requires confirmation at histology, given the tendency to find the lesions distant from the uterus. However, histology of a benign smooth muscle tumor distant from the uterus in a patient with a history of laparoscopic myomectomy with morcellation should increase the suspicion of a parasitic leiomyoma [10]. This is also important in order to differentiate this benign condition from other malignant abdominal conditions.

The initial investigation, just as in cases of a patient presenting with a pelvic mass, should therefore include a pelvic ultrasound. MRI may further help in distinguishing benign leiomyomas from other solid pelvic and abdominal tumors [10]. The nondegenerated myomas are typically hypointense on T2-weighted images and isointense on T1-weighted images. The difficulty in differentiating the benign leiomyoma from the malignant leiomyosarcoma emphasizes the need for a thorough clinical history and histological diagnosis. Our patient had a pelvic scan and an abdominopelvic MRI.

Measurement of tumor markers such as CA 125, α-fetoprotein, or CA19-9 may be done, though they may be falsely elevated with occasional ascites [10]. Our patient had no clinical or radiological ascites, and given her previous history of laparoscopic myomectomy, we did not consider measuring the tumor markers, because the mass was unlikely to be malignant. However, in cases of suspected malignancy such as leiomyosarcoma, tumor markers may be useful in monitoring therapy and follow-up, especially if initially elevated [15].

Treatment involves excision of the parasitic myoma. This would be achieved through laparoscopy with good outcomes or through laparotomy, especially in cases of large myomas or suspected malignancy. In our patient, excision was performed through a laparotomy. Whenever possible, a multidisciplinary approach should be used, especially when the parasitic myoma is near other organs [10]. This was not necessary in our patient, however, because the myoma was on the anterior abdominal wall.

To reduce the risk of developing parasitic leiomyomas following power morcellation, various alternatives have been suggested [1, 9]. The morcellation can be performed in a containment bag. This reduces spread of the morcellated fragments. However, when malignancy is suspected, morcellation should be avoided. Large specimens can be retrieved from the abdominal cavity by bisecting or by cutting in small portions that can then be removed though colpotomy or minilaparotomy. In addition, it is essential to carefully check the abdominal cavity after morcellation to avoid leaving myoma fragments [2, 4, 8], though this may still be insufficient to remove all tissue fragments [9].

## Conclusions

Parasitic leiomyomas are a rare late complication of power morcellation following laparoscopic myomectomy or hysterectomy. Most patients present with an abdominal/pelvic mass and may need surgical excision to relieve the symptoms. Care should be taken during power morcellation to prevent excessive fragmentation of the tissues, some of which may become implanted and persist to form parasitic myomas. Moreover, effort should be made to retrieve all myoma fragments by carefully checking the abdominal cavity. Whenever possible, the morcellation should be done in a containment bag.
